# Novel Magnetic Polymeric Filters with Laccase-Based Nanoparticles for Improving Congo Red Decolorization in Bioreactors

**DOI:** 10.3390/polym14122328

**Published:** 2022-06-08

**Authors:** Diana C. Sotelo, Nancy Ornelas-Soto, Johann F. Osma

**Affiliations:** 1 Department of Electrical and Electronic Engineering, Universidad de los Andes, Bogotá 111711, Colombia; dc.sotelo10@uniandes.edu.co; 2 Laboratorio de Nanotecnología Ambiental, Escuela de Ingeniería y Ciencias, Tecnológico de Monterrey, Monterrey 64849, Mexico; ornel@tec.mx

**Keywords:** decolorization, Congo Red dye, nanocomposites, magnetite, laccase, magnetic field, biofilters

## Abstract

In this work, five different magnetic biofilters, containing magnetic nanoparticles (142 nm), immobilized laccase on nanoparticles (190 nm) and permanent magnetic elements, such as neodymium magnets and metallic meshes, were designed, manufactured and tested. The five types of filters were compared by measuring the decolorization of Congo Red dye inside bioreactors, the half-life of the filters and the amount of magnetic nanoparticle and enzyme lost during multiple cycles of operation. Filters containing laccase immobilized on magnetite (Laccase-magnetite), permanent magnets and metallic mesh presented the highest Congo Red decolorization (27%) and the largest half-life among all types of filters (seven cycles). The overall dye decolorization efficiencies were 5%, 13%, 17%, 23%, and 27% for the paper filter, paper filter with magnetite, paper filter with Laccase-magnetite, paper filter with Laccase-magnetite with magnets and paper filter with Laccase-magnetite with magnets and metallic mesh, respectively. Although the highest losses of magnetite occurred when using the filters containing magnets (57 mg), the use of permanent magnetic elements in the filters increased the half-life of the filter three-fold compared to the filters without enzymatic properties and two-fold compared to the filters with Laccase-magnetite. Results indicate that the novel use of permanent magnetic elements improved the nanoparticle retention in the filters and promoted the mass transfer between the dye and the biocatalyst to enhance wastewater treatment.

## 1. Introduction

Wastewater treatment systems tend to mimic natural biological, physical, and chemical methods [1]. Technologies used to treat wastewater can be classified into two categories, extensive and intensive. Extensive technologies tend to be less energy intensive but may present efficiency rates. Intensive technologies (e.g., activated sludge and other biotechnologies) have high biochemical reaction rates due to the addition of oxygen or application agitation [1,2].

Recently, the use of membrane technologies in bioreactors has emerged as a preferred choice for recovering water from wastewater streams [2], since membranes can retain pollutants of different origins and chemical nature [3]. In addition, membranes can be improved by functionalizing nanoparticles, such as silver, or enzymes, such as laccase or lipases [2]. For example, silver nanoparticles provide disinfection and inactivation of microorganisms [4], and laccases can oxidize multiple substrates found in the stream [5].

Laccase-based technologies have a wide array of applications in the textile industry, as they can be used for the biodegradation of synthetic dyes [6]. Textile dyeing is the second largest polluter of water [1], using 2000 gallons of water to fabricate a typical pair of jeans. Given the current scenario, the fashion industry will use up to a quarter of the world’s carbon budget by 2050 [7]. According to the World Bank report of 2019, the treatment and dyeing of textiles are responsible for up to one fifth of industrial water pollution globally, including the emission of 72 toxic chemicals (e.g., heavy metals, Glauber salt, oils, dyes, sulfur, among others) into the water supplies. In this sense, the treatment of water effluents from the textile industry has become imperative to counter their environmental impact [7,8].

About 70% of the dyes present in textile effluents contain azo (N = N) chromophores, which are thought to be responsible for fluctuations in the chemical oxygen demand, color, salinity and pH [9]; thus, numerous research efforts have focused on the development of advanced oxidation processes (AOPs) to treat these effluents. AOPs are oxidative methods based on the generation of intermediate radicals, mainly hydroxyl radicals (OH), that have been successfully applied in wastewater treatment to degrade many organic compounds [10]. However, biological processes are gaining tremendous attention in various fields due to the lower chemical requirement, cost-effectiveness and eco-friendly operating techniques [11]. In this sense, oxidoreductase enzymes, such as laccases, capable of carrying out these oxidation processes without the need for harsh chemical compounds have become an attractive alternative [12]. Nevertheless, enzyme-based wastewater treatment methods tend to be expensive due to the lack of reusability and purification processes [9,12]. Therefore, enzyme-immobilization has gained attention to overcome the reusability and stability issues, by generating strategies to recover the immobilization substrate and thus, recovering the enzyme for further use.

Several functionalization and immobilization strategies have been proposed to improve enzyme stability and resistance to environmental changes, such as the use of surface fillers, and polymers, among others [13,14]. Pertaining to immobilization processes for surface coverage, protein-protein interactions (e.g., electrostatic forces, hydrogen bonding) are favored over surface-protein interactions (e.g., bonding, chemical or physical adsorption) and the appropriate immobilization method will be chosen depending on conditions, such as pH, temperature, reaction time, among others [14,15].

Immobilized enzymes have been integrated into different types of systems to improve their modification or processing capabilities and increase enzyme stability and thus, improve the operability of laccases in practice [16,17]. There are different types of laccase immobilization, such as adsorption, mesh embedding, microencapsulated embedding, covalent binding, self-immobilization and two step combination. Covalent binding immobilization has advantages, such as the strong binding force between laccase and the surface carrier, broad applications, and long-term continuous use [18]. When laccase is immobilized into filters, these biofilters can have a great impact on bioremediation due to their high performance [19,20].

There are numerous treatment methods, based on adsorption methods, for the effective treatment of wastewater loaded with organic dyes [21,22]. Furthermore, different types of nanoparticles that promote the contact between the adsorbent and adsorbate, such as ferrites, can also reduce the equilibrium time of the reactions [21]. The adsorption processes of dye-containing waters, combined with magnetic compounds, are highly favored as additional active adsorption sites are generated [21].

Laccase biofilters for wastewater treatment can be included in a continuous operation or a batch operation [23]. The use of biofilters in continuous flow reactors allows for the permanent treatment of wastewater with a slower reaction time than in batch reactors [23,24]. Thus, improving the contact time and interaction between the pollutants and the laccase of the biofilters is highly desirable to increase the wastewater treatment capacity of continuous flow bioreactors.

In this study, we focused on the immobilization of laccase on magnetic nanoparticles, which allows the manipulation of the enzymes through the interactions between the nanoparticles and provoked magnetic fields [25]. In addition, the combination of these two technologies (immobilization and magnetic compounds) produces more powerful treatments. Other authors reported that the enzymatic activity was maintained or increased after immobilization on magnetite, even after several treatment cycles [26,27,28]. Other studies showed that water treatment, in terms of removal or decolorization, was improved by applying an external magnetic field [27]. In the same line, some industrial applications are limited due to the high production costs, low stability, and poor reusability of free laccases, so laccase immobilization on materials, such as magnetite provides a general approach to overcome the above drawbacks [28]. Nevertheless, no previous studies involving magnetic nanoparticles, enzymes, and permanent magnetic fields have been reported for the treatment of wastewaters or dye degradation.

Herein we designed five different types of magnetic filters for wastewater treatment that seek to improve the retention of laccases through permanent magnetic fields. Artificial wastewater loaded with Congo Red was treated in continuous flow bioreactors using the different types of magnetic filters. Our findings suggest the treatment of paper-based filters with laccase immobilized onto magnetite nanoparticles, and the addition of permanent magnetic elements in the filter provides a suitable approach to achieve more efficient laccase dye decolorization than results obtained with similar filters.

## 2. Materials and Methods

### 2.1. Materials and Reagents

All materials were used as received and with no further purification. Filter paper No. Cat 1005 110, 110 nm diameter (Whatman), 2,2′-azino-bis (3-ethylbenzothiazoline-6-sulfonic acid) diammonium salt (ABTS), glutaraldehyde (25%), sodium hydroxide (NaOH) (98%), tetramethylammonium hydroxide (TMAH) (25%), γ-Aminopropyl-triethoxysilane (APTES) (98%), hydrochloric acid (HCl), potassium dihydrogen phosphate (KH_2_PO_4_) and potassium hydrogen phosphate (K_2_HPO_4_) were purchased from Merck (Darmstadt, Germany). A Bradford Protein Assay kit was purchased from Bio-Rad (Hercules, CA, USA). Polymethylmethacrylate (PMMA) sheets of 2 mm thickness were obtained from local distributors (Bogota, Colombia). Iron (II) chloride tetrahydrate (98%) (FeCl_2_·4H_2_O), Iron (III) chloride hexahydrate (97%) (FeCl_3_·6H_2_O), were obtained from PanReac AppliChem (Madrid, Spain). Congo Red was purchased from ABC laboratorios (Bogota, Colombia).

### 2.2. Laccase Production and Purification

Laccase enzyme was produced using *P. Sanguineus* CS43 grown on a tomato medium as previously described [29]. Mycelia, from the culture supernatant, was filtered using two filters in serial configurations (i.e., 0.5 mm pore size and 0.2 mm pore size). Ultrafiltration was carried out using a 10 kDa cut-off membrane. Two laccase isoforms (Lac I and Lac II) were obtained with a high amino acid similarity (91%), and a high thermostability at 50 °C and 60 °C, with a half-life of 277.7 h and 18 h for Lac I and 35.8 h and 2.25 h for Lac II, and molecular weights of 68 kDa and 66 kDa, respectively [29,30].

### 2.3. Enzyme Characterization

The stability of free and immobilized laccase (Laccase-magnetite) was examined at pH values of 2.0, 3.0, 4.0, 4.5, 5.0, 6.0 and 7.0 at 25 °C using a phosphate-citrate buffer solution. The enzymatic activity of free and immobilized laccase was determined spectrophotometrically as described by Moilanen et al. [31] at 436 nm using a GENESYS 10 S UV-Vis v4.004 2L5R078128 (Thermo SCIENTIFIC, Waltham, MA, USA). One activity unit was defined as the amount of enzyme that oxidized 1 µmol of ABTS per min. Activity was expressed in U/L.

Laccase concentration was determined by the Bradford method [32] using a Genesis 10 S spectrophotometer (Thermo Fisher Scientific, Waltham, MA, USA) at a 595 nm wavelength. In this case, 100 µL of the sample was mixed with 200 µL of Bradford reagent. After 15 min the absorbance at 595 nm was measured. Laccase concentration was determined using a BSA standard curve. All measurements were carried out in triplicate.

### 2.4. Synthesis of Magnetite Nanoparticles

Magnetite nanoparticle synthesis was performed via coprecipitation by mixing 20 mL of 1 M FeCl_2_ and 20 mL 2 M FeCl_3_ under agitation at 1500 RPM and 90 °C. Then, 40 mL of 8 M NaOH and 40 mL 2% (*v*/*v*) of TMAH were added to the mixture for 3.5 h at a flow rate of 12 mL/h. The obtained nanoparticles were purified using magnetic separation, with the assistance of a strong magnet continuously attached to the surface of the container. The next step consisted of washing the nanoparticles thoroughly, where 2% (*v*/*v*) TMAH was added, and finally sonicated for 100 min using a VibraCell sonication system (Newtown, CT, USA). Afterward, the synthesized magnetite nanoparticles displayed an average hydrodynamic diameter of 88.59 nm with a polydispersity index of 0.182 as determined by DLS analysis with the aid of a Zetasizer Nano ZS, (Malvern, PA, USA). The morphology of those nanoparticles was analyzed as previously described by Lopez-Barbosa et al., (2020) [9].

### 2.5. Laccase Immobilization

**Figure 1:**
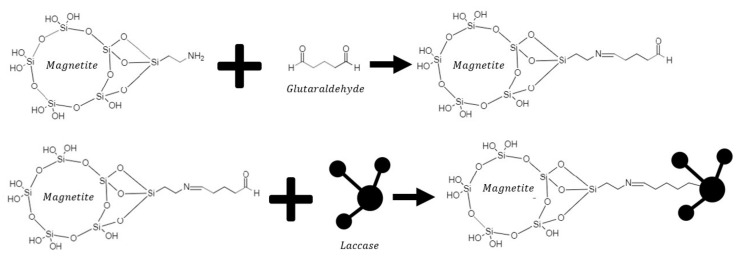
Reaction mechanism for enzyme immobilization. Upper panel shows the silanization with APTES and later crosslinking of NH_2_ terminal groups with the aid of glutaraldehyde. Bottom panel shows the conjugation of laccase by forming imine bonds with the pre-activated magnetite nanoparticles.

Laccase immobilization on magnetite nanoparticles was carried out via surface functionalization using amino-silane (APTES), glutaraldehyde and then the enzyme (Figure 1). First, 0.6 g of wet magnetite in 8 mL of Milli-Q water was sonicated for 20 min. After that, 200 µL of TMAH was added and sonicated for 10 min; 500 µL of 0.5 M NaOH was added and mixed manually for 2 min until a pH above 10 was reached; 250 µL of 98% (*v*/*v*) APTES was added, the solution was sonicated for 10 min, and afterward washed with Milli-Q water. Once the particles were silanized, 100 µL of 50% (*v*/*v*) glutaraldehyde was added and mixed with a vortex at room temperature for 1 min, and left static for 30 min. The sample was washed thoroughly five times with Milli-Q water to remove the excess of unbonded glutaraldehyde, and then, nanoparticles were resuspended in 5 mL of Milli-Q water and 200 µL of 50,000 U/L Laccase was added. Then, the functionalized magnetite (Laccase-magnetite) was left overnight under static conditions at room temperature. Finally, Laccase-magnetite was washed thoroughly two times with Milli-Q water to remove the excess of unbonded laccase and stored at 4 °C.

Laccase immobilization on magnetite (Laccase-magnetite) was confirmed by Fourier transform infrared spectroscopy (FT-IR) on an A250/D FTIR-ATR (Bruker, Billerica, MA, USA), along with enzymatic activity, and immobilization efficiency. Immobilization efficiency was quantified by measuring protein concentration using the Bradford method, as previously described. A schematic of the reaction mechanism of immobilization is shown in Figure 1.

Zetasizer Nano ZS, (Malvern, PA, USA) equipment was used to determine the size and size distribution of the magnetite nanoparticles (MNPs). Samples were prepared by diluting the nanoparticles to 3% (*w*/*v*) in 1 mL of Milli-Q water and TMAH prior to the analysis. Nanoparticles morphology was analyzed as previously described by Lopez-Barbosa et al., (2020) [25].

### 2.6. Nanoparticle Immobilization on Filters

One hundred milligrams of either magnetite or Laccase-magnetite nanoparticles in solution were deposited on polymeric filters made of paper within the PMMA rings. The mass of nanoparticles added per filter was 14.08 mg/cm^2^. In the case of filters with Laccase-magnetite, each filter contained 568.7 µg and 21.9 U of laccase enzyme, representing 80.5 µg/cm^2^ and 3.1 U/cm^2^ of the enzyme per area of each filter.

### 2.7. Bioreactor Setup and Decolorization Measurements

**Figure 2:**
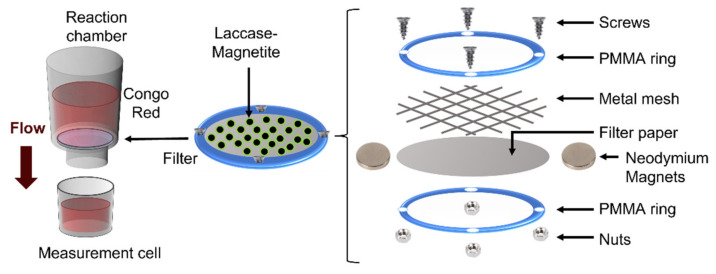
Filters schematic. The filters consisted of a reaction chamber and a measurement cell. The reaction chamber contained a paper-based biofilter with magnetite or magnetite with immobilized laccase molecules as biocatalysts and 50 mL volume of artificial wastewater.

Five different filters were designed and tested inside bioreactors to examine their decolorization properties. The setup of each type of filter consisted of the use of filter paper, pressed by two PMMA rings. Depending on the filter, the presence of magnetite or Laccase-magnetite was included as previously described. In addition, permanent magnetic elements, such as 380 mT neodymium magnets and metallic mesh, were incorporated into some filters. Therefore, the study contemplated five types of filters described as filters with just filter paper (P), P filter with magnetite nanoparticles (N), P filter with Laccase-magnetite (L), P filter with Laccase-magnetite with magnets (M), P filter and Laccase-magnetite with magnets and a metal mesh (G) as shown Figure 2. Each filter had two PMMA rings, which were designed with an external diameter of 40 mm, an internal diameter of 30 mm and four holes of 3 mm diameter where the 1/8“screws were located to secure the filter (Figure 2 right side). The contact area of the filter with the wastewater was 7.1 cm^2^.

The filter was secured to the bottom of the reaction chamber through a screw system to avoid leakages. The complete setup consisted of a reaction chamber, containing a filter, and a measurement cell as shown in Figure 2 (left side). The reaction chamber, the top part of the bioreactor, contained the untreated water solution prepared from the dilution of 60 mg/L Congo Red in milli-Q water at pH 6.0, which was kept in the dark and at room temperature until tested. Congo Red dye characteristics are shown in Table 1. On the bottom part of the bioreactor, the measurement cell collected the treated water after passing through the filter.

Congo Red decolorization was calculated by measuring the absorbance peak at 495 nm wavelength and comparing the initial and subsequent absorbance in terms of percentage. The amount of magnetite lost was established by weighing the filters before and after completing three treatment cycles. The amount of laccase lost during each filtration process was calculated by the Bradford assay as previously described.

Each treatment cycle started with 50 mL of fresh untreated water in the reaction chamber and finished when all the untreated water passed through the filter and was collected in the measurement cell. Then, samples were collected to determine the decolorization percentage, as well as magnetite and laccase lost during the filtering process. Three cycles were carried out for each type of filter. All experiments were carried out in triplicate.

### 2.8. Confocal Laser Microscopy of the Nanoparticles Added on the Filters

Confocal laser scanning microscope FLUOVIEW FV1000 (Olympus, Tokyo, Japan) was used to evaluate magnetite and Laccase-magnetite. Confocal laser scanning microscope fluorescence images of magnetite nanoparticles and Laccase-magnetite nanoparticles were obtained and examined with 559 nm and 633 nm lasers as light sources at 20× magnification.

## 3. Results

### 3.1. Impact of pH Changes on the Enzymatic Activity

**Figure 3:**
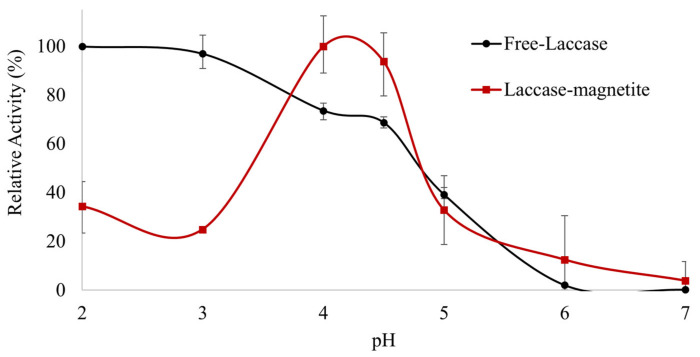
pH profile of free and immobilized laccase on magnetite nanoparticles (Laccase-magnetite).

The impact of pH on the enzymatic activity was evaluated as shown in Figure 3. The optimal activity for the free laccase was evidenced under acidic conditions, especially at pH 2. Several optimal values between pH 2 and 4 were reported by other studies [33,34]. For pH values of 5.0, the activity decreased to less than 50% and at pH 6.0 the activity decreased almost completely. In the case of the Laccase-magnetite, activity reached a peak at pH 4.0, followed by a considerable activity decrease for pH values of 6.0 and above. The change in optimal pH for Laccase-magnetite to higher values of relative activity is probably due to a different concentration of the H+ and OH− ions between the support matrix and the bulk solution [35,36].

### 3.2. Immobilization and Characterization of Magnetite and Laccase-Magnetite

**Figure 4:**
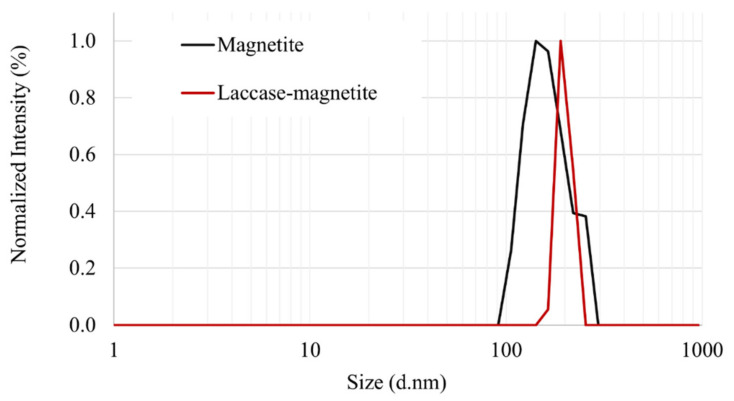
Zetasizer results of magnetite nanoparticles and Laccase-magnetite nanoparticles.

Laccase immobilization efficiency was determined as 98% according to laccase concentration, and 90% according to laccase activity. Immobilization was confirmed using FTIR and confocal microscopy. Zetasizer showed that the synthesized magnetite nanoparticles presented a narrow peak at 142 nm, while the Laccase-magnetite presented a peak at 190 nm as shown in Figure 4. According to these results, it is possible to determine that there is a difference in terms of the size of the magnetite and the functionalized magnetite with the enzyme (Laccase-magnetite), the latter having a larger size distribution, with an average of approximately 50 nm larger than the hydraulic diameter of magnetite. In addition, magnetite presents a slighter broader size distribution.

**Figure 5:**
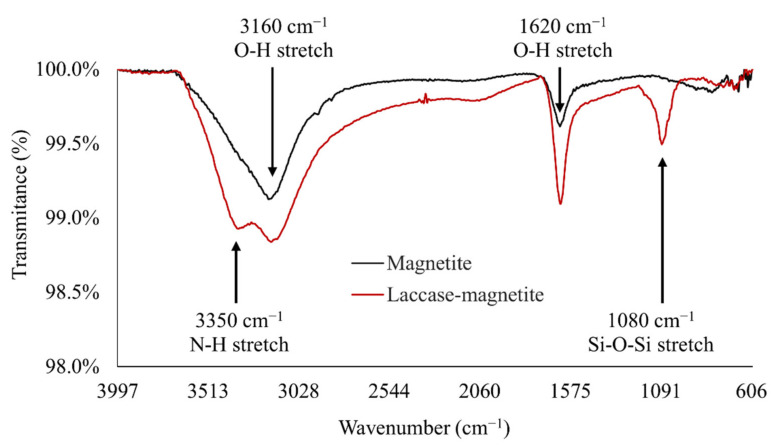
FTIR analysis of magnetite nanoparticles and Laccase-magnetite. The presence of peaks at 1620 and 3160 cm^−1^ corresponds to the OH stretch from the magnetite surface. The presence of the peak at 1080 cm^−1^ corresponds to the O-Si-O bond and corroborates effective silanization. The immobilization of laccase was confirmed by the presence of the amide I peak at around 3350 cm^−1^.

Silanization of silica nanoparticles was confirmed by FTIR analysis. Figure 5 shows the average FTIR spectra of magnetite and Laccase-magnetite. Two bands of ν (Fe-OH), identified at 1620 and 3160 cm^−1^, represent the vibration of the hydroxyl groups on the magnetite surface, as reported in other studies [37], particularly, the peak at 3160 cm^−1^ is attributed to single bond OH [38]. Therefore, it is possible to affirm that these peaks correspond to the surface of the magnetite. In the case of Laccase-magnetite, there are two significantly different peaks compared to the spectrum of magnetite, the first one observed at 3350 cm^−1^ was attributed to the NH stretch present in the laccase amides [39]. The second peak was observed at 1080 cm^−1^ characteristic of the Si-O-Si bond. This band confirms the silanization of the magnetite surface as part of the functionalization process for enzyme immobilization [9].

**Figure 6:**
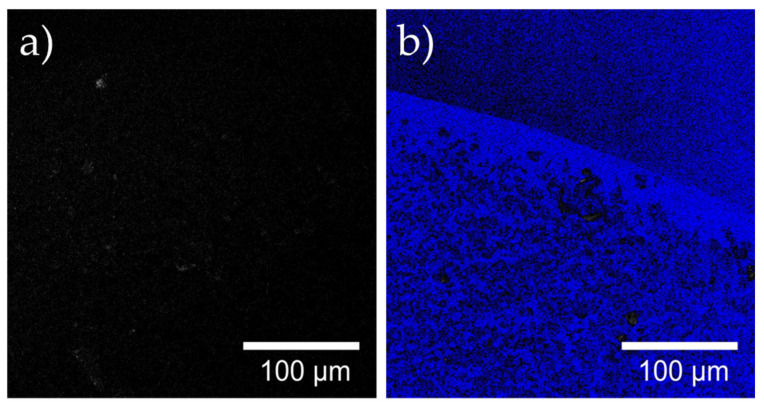
Confocal images using two excitation lasers 559 nm and 633 nm at 20× of (**a**) magnetite and (**b**) Laccase-magnetite. Images combined the optical image of both excitations.

Successful immobilization of laccase molecules was also demonstrated by direct observation of magnetite and Laccase-magnetite under confocal microscopy (Figure 6). The confocal images show a difference between both types of nanoparticles, where laccase distribution is relatively homogeneous throughout the material. Both samples were excited with a 553 nm laser that stimulates in the range of 570–670 nm and later, exposed to a 633 nm laser that stimulates in the range of 660–730 nm. Images taken from both excitation sources were overlapped for each type of nanoparticles (magnetite and Laccase-magnetite) and a significant difference was evidenced in the fluorescence response, where Laccase-magnetite nanoparticles showed specific fluorescence attributed to the presence of laccase.

### 3.3. Nanoparticle Immbolization on Filters

**Figure 7:**
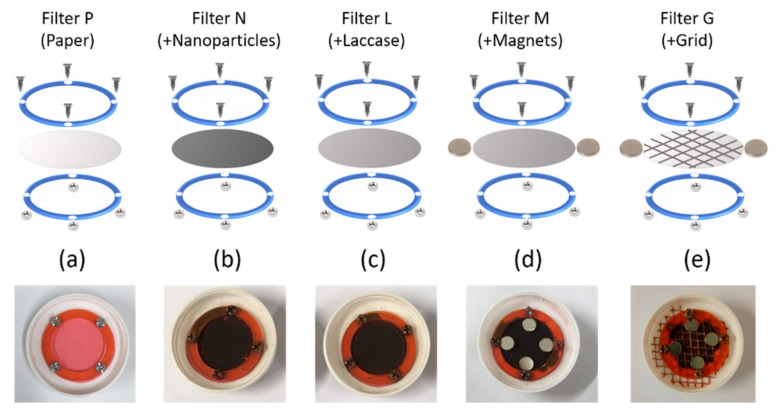
Filters design. The evaluated filters were: (**a**) Filter P: filter paper without modification, (**b**) Filter N: Filter P with magnetite, (**c**) Filter L: Filter N with Laccase-magnetite, (**d**) Filter M: Filter L with magnets and I Filter G: Filter M with metal mesh (**e**).

Filters were assembled and used as shown in Figure 7. P filters only consisted of filter paper, with a red coloration due to the treatment of Congo Red solutions. Filter N and L contain only magnetite or Laccase-magnetite nanoparticles, respectively. Filter M includes Laccase-magnetite and four permanent neodymium magnets, and filter G has Laccase-magnetite, four permanent magnets and a metallic mesh. All filters were assembled to ensure the same fit once assembled in the bioreactors, and no leakages could be attributed to changes in the Congo Red degradation.

### 3.4. Bioreactor Setup and Decolorization Measurements

**Figure 8:**
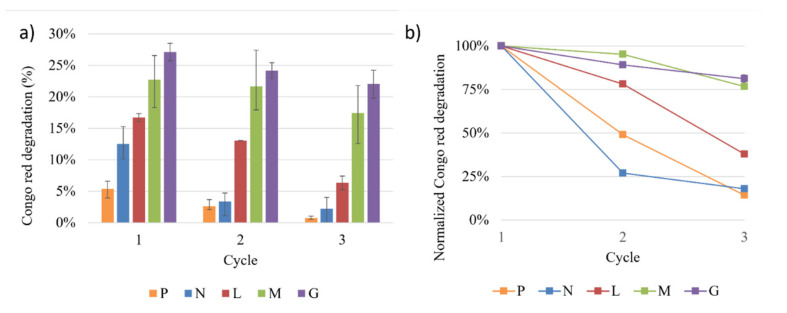
Decolorization measurements of Congo Red using the different filters: filter paper (P), P filter with magnetite nanoparticles (N), P filter with Lac-case-magnetite (L), P filter with Laccase-magnetite with magnets (M), P filter and Laccase-magnetite with magnets and a metal mesh (G). (**a**) decolorization per cycle, (**b**) normalized decolorization per cycle.

Measurements were recorded in continuous flow to determine the Congo Red decolorization efficiency of each type of filter at different working cycles. Figure 8 shows the percentage of decolorization of P, N, L, M and G filters after every cycle.

Each cycle took approximately 45 min to 60 min to finish the filtering of 60 mL of untreated water. As shown in Figure 8, the highest decolorization percentage for all cycles was obtained with the Laccase-magnetite filters (L), Laccase-magnetite with magnets filters (M) and Laccase-magnetite with magnets and mesh filters (G). As expected, the enzyme-containing setups were more effective than those only based on porous materials (i.e., paper filter, magnetite). Our findings showed that the effectiveness of the filters decreases as the number of cycles increases. It is possible to affirm that adding certain elements that improve magnetite retention (i.e., magnets and metallic mesh), significantly improves the decolorization efficiency carried out by the laccase molecules found on the surface of the nanoparticles. However, none of the evaluated treatments showed a decolorization percentage higher than 30%.

Figure 8b shows that the P and N filter reached their half-life in the second cycle, while the filters containing Laccase-magnetite presented a higher half-life. The L filter reached its half-life in the third cycle, and the filters that used magnets (M and G) presented a significant increase in their half-life, i.e., more than the three tested cycles. In order to estimate their half-life, a linear regression was carried out, showing a half-life of six cycles for the M filter (R^2^ of 0.896) and seven cycles for the G filter (R^2^ of 0.991).

**Figure 9:**
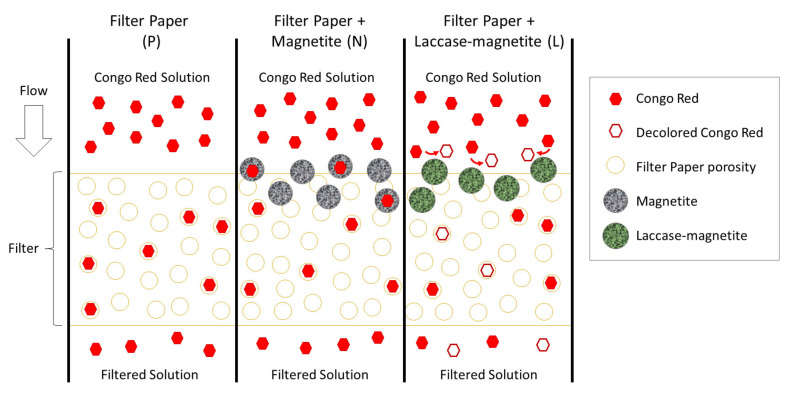
Interaction between the filter material and Congo Red dye molecules diagram.

Different processes may have occurred during the decolorization process of the Congo Red for every type of filter. Figure 9 describes the most probable mechanisms within the filter. Adsorption processes of the Congo Red molecules may have occurred when in contact with the porosity of the filter paper. This was probably the main mechanism inside P filters. In filters containing magnetite nanoparticles, additional adsorption mechanisms took place between the porosity of the magnetite and the dye (N filters). In those filters containing immobilized laccase on the magnetite, an additional enzymatic treatment took place. In those filters (L, M and G), laccase degraded Congo through oxidation leading to species with nitrification of the NH_2_ group and a loss of the SO_3_ group, as previously reported by other authors [40].

### 3.5. Loss of Magnetite and Laccase-Magnetite

**Figure 10:**
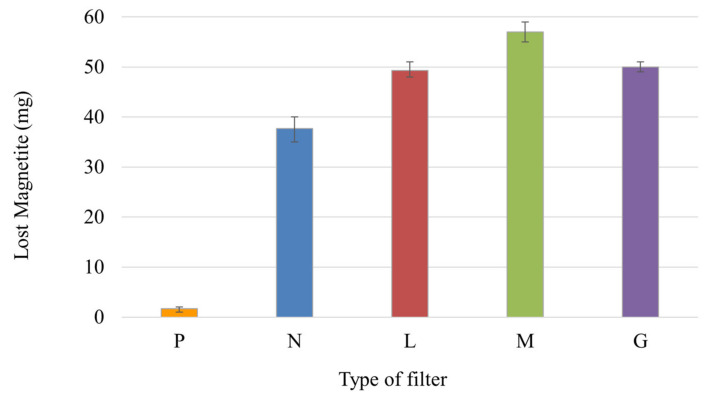
Lost magnetite using the different filters P, N, L, M and G during three cycles of operation.

Nanoparticle loss was quantified for each type of filter after successive cycles of operation (Figure 10). Despite the observed losses of magnetite in filters M and G, it is possible to state that the distribution of the magnetic field helps the process to be more effective. Therefore, there is no evidence of a correlation between the mass of magnetite that was lost and the effectiveness of the decolorization of the Congo Red dye. As shown in Figure 10, it can be stated that the type of filter that showed the lowest nanoparticle losses was the one modified with magnetite (N filter) with nearly 38 mg (38%) of lost nanoparticles after three cycles of operation. The L and G filters presented about the same amount of lost magnetite accounting for nearly 50 mg (50%), while the highest nanoparticle loss was shown by the M filter with approximately 57 mg (57%). We can assume that the lost magnetite value in the case of the paper filter corresponds to the error of the measurement method, as the P filter did not contain nanoparticles.

**Figure 11:**
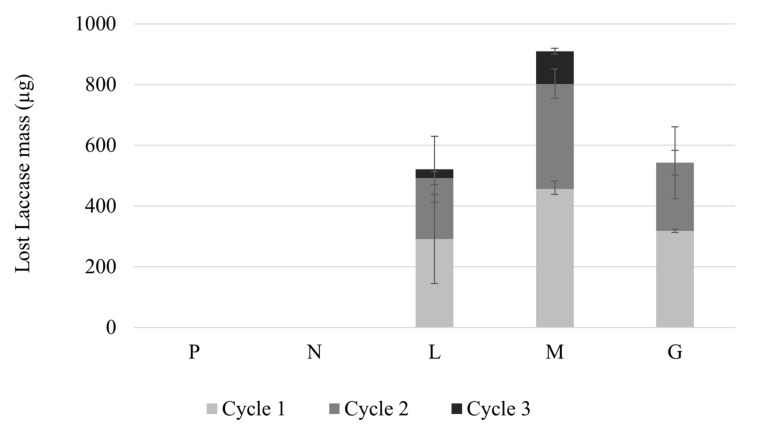
Lost Laccase per cycle using the different filters P, N, L, M and G.

Enzyme concentration was measured in the treated water after each Congo Red decolorization cycle using the Bradford method; therefore, enzyme mass lost was calculated for each type of filter and cycle of operation. The P and N filter did not contain laccase in their composition; therefore, their losses of the enzyme were null. Figure 11 shows that the amount of lost enzyme decreased in each cycle for all types of filters. The type of filter that presented the least retention of laccase was the one modified with Laccase-magnetite with magnets (M filter), as was evidenced by the loss of magnetite in Figure 10.

Figure 10 and Figure 11 show an existing correlation between the quantified lost magnetite and the quantified lost laccase (L, M and G filters), showing that enzyme loss is highly dependent on the lost magnetite, suggesting that the laccase was bound to the lost magnetite. However, there is no correlation between the lost laccase per cycle and the half-life presented for each type of filter, indicating that there are additional phenomena taking place in the reaction. Loss of efficiency in the enzyme-based filter can be attributed to enzyme leakage, mechanical damage from successive reactions, rinsing processes [41], or the continuous flow of the untreated water; however, mass transfer limitations can also result in lower activity [42]. In our study, the efficiency of the enzymatic filters declined over treating cycles; thus, affecting the efficiency of the decolorization over time. This can probably be attributed to the mechanical damage derived from successive reactions [41]. Therefore, the use of permanent magnetic elements, such as neodymium magnets and the metal mesh may be enhancing mass transfer between the Lacasse-magnetite and the Congo Red, for this reason, despite the loss of Laccase-magnetite, the life-time of these filters (M and G filters) increased considerably compared to those without permanent magnetic elements (L filters). However, it is necessary to consider the limitations of mass transfer phenomena and that the results may vary between a continuous process and measurements made in a short and established time. In this study, it is assumed that there is sufficient time for the Congo Red to diffuse into the filter membrane and react with the immobilized enzyme.

## 4. Discussion

Laccases are polyphenol oxidase enzymes capable of oxidizing different types of phenol and non-phenol compounds using oxygen molecules as electron acceptors and transforming them into water [9,43]. This effect was demonstrated by measuring the water that passed through the filter using spectrophotometry. These results confirm that by combining laccase with the magnetite nanoparticles and with a permanent magnetic field, the percentage of dye decolorization increased compared to only the use of magnetite or Laccase-magnetite nanoparticles in the filter. This behavior has been previously described for systems where immobilized laccase enzyme from *Streptomyces cyaneus* was applied for laccase-coated yeast cell walls and its efficiency vs. a native polymer [13], and for systems where immobilized lipase enzyme from *Candida rugosa* was applied for the lipolytic activity on gold nanoparticle surfaces [44].

The first treatment cycle presented the greatest enzyme loss in mass, which affected the following treatment cycles, reducing the overall decolorization percentage. The cycle with the greatest loss of laccase was the first version of magnetic field modification (filter with Laccase-magnetite and magnets), this filter had the greatest loss of magnetite in weight. This can be attributed to the agglomeration of magnetic particles near the magnets, causing an uneven distribution over the surface of the paper filter, and therefore, a less active surface area available for wastewater treatment.

The filters or treatments that presented the lowest levels of dye removal were paper filters (P filters) and magnetite nanoparticles (N filters) (5% and 13%, respectively). On the other hand, the filters that presented the highest levels of dye removal were Laccase-magnetite with magnets (M filters) and Laccase-magnetite with magnets and a metal grid (G filters) with 23% and 27% of decolorization, respectively. The results suggest that the filters with magnets and metallic mesh create an appropriate distribution of the magnetic field that helps in the interaction between the dye and the Laccase-magnetite. However, all filters that incorporated Laccase-magnetite (L, M and G filters) presented higher decolorization rates than those without enzymes. The use of permanent magnetic elements, such as magnets and the metal mesh in the filters (M and G) increased the half-life of the filter three-fold compared to the filters without enzymatic properties (P, N filters) and two-fold compared to filters with Laccase-magnetite (L filters). This can probably be attributed to the effect of the permanent magnetic elements in the mass transfer between the Lacasse-magnetite and the Congo Red.

Some degradation methods of azo dyes reach efficiencies of 75–81% [45,46,47]; however, in the case of treatments for the decolorization of diazo dyes, such as Congo Red, the reported removal percentages are similar to those obtained in this study, between 30 to 40% [9]. Although the removal percentage is lower than those reported in other studies where filters with laccase immobilized in magnetite were also evaluated [9], it is important to mention that the type of membrane and the treated volume used were different and that in this case, magnetic fields are used to improve retention. Other studies use bacterial strains instead of enzymes [48] and obtain high percentages of decolorization, but these studies must be carried out for longer periods of time [47,49] than those evaluated in this study.

Not many studies using permanent magnets for wastewater treatment are reported. Most of them base their reports on the calculations and estimations of the magnetic field created by the magnets [50]. Magnetic systems have also been used in the food industry and in water treatment technology, but without immobilized enzymes in the filters [51]. Using such reported treatment methods, the researchers achieved successful results in which all the organic dyes tested were removed with an efficiency of more than 80% by a combination of magnetic separation and magnetic seeding [52]. Nevertheless, the use of enzymatic magnetic nanoparticles, with the application of permanent magnetic fields inside bioreactors under continuous flow operation has never been tested before this study.

## 5. Conclusions

Although this study did not include the modeling of the distribution of the permanent magnetic field, and Congo Red dye was used as a model molecule and no real wastewater samples were included, we report the use of paper-based filters modified with magnetite nanoparticles and permanent magnetic elements. Two types of nanoparticles, namely, magnetite and Laccase-magnetite, were incorporated into the filters as the active component for the decolorization of Congo Red dye, while magnetic elements were added to improve the treatment capabilities of the filters. According to our results, the proposed approach presented clear benefits in the Congo Red decolorization, with the best decolorization achieved for combinations of filters with Laccase-magnetite nanoparticles in the presence of magnets and a metal grid. In addition, the half-life of the filters with magnets and metal grid severely increased three-fold compared to the filters without enzymatic properties and two-fold compared to filters with Laccase-magnetite. This was attributed to the effect of the magnetic elements in the mass transfer phenomena between Congo Red and the Laccase-magnetite.

## Figures and Tables

**Table 1:** Main features of Congo Red dye used to study the decolorization performance of the filters [9]. Azo-bonds labeled in blue circles.

Dye	λmax (nm)	Color Index Number	Color Index Name	Structure
Congo Red	495	22120	Direct Red 28	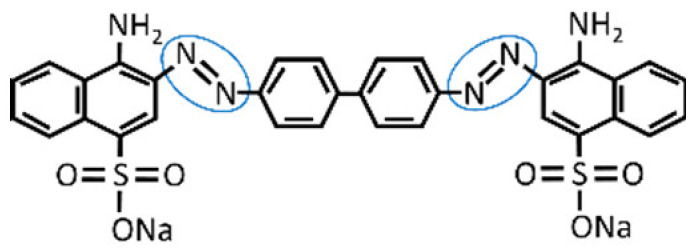

## Data Availability

The data and contributions presented in the study are included in the article. Further inquiries can be directed to the corresponding author.
